# Cultural Diversity and Mental Health Care: A Case Study

**DOI:** 10.1192/j.eurpsy.2024.1084

**Published:** 2024-08-27

**Authors:** M. R. Soares, M. P. Cameira, P. Abreu, M. Pereira

**Affiliations:** Lisbon Psychiatric Hospital Centre, Lisbon, Portugal

## Abstract

**Introduction:**

In today’s world of global migration and cultural diversity, mental health care grapples with persistent challenges. Despite efforts to promote cultural competency and person-centred approaches, it’s vital to delve into the issues surrounding cultural differences and linguistic diversity in mental health care. This exploration highlights the complexities where culture, language, and healthcare intersect (Brisset et al., 2014; Desai et al., 2021).

**Objectives:**

Our aim is to analyse cultural and linguistic barriers in mental health care for migrants and assess their impact on access and quality of care.

**Methods:**

Literature review, drawing from sources such as PubMed, ResearchGate, and Google Scholar. This review will be framed around the case of a 34-year-old man from Bangladesh, who has been residing in Lisbon for a year. His clinical presentation includes depressive symptoms, disorganized behaviour, and psychotic manifestations, such as persecutory delusions. He does not speak Portuguese or English, thereby limiting his access to essential mental health treatment services. Through this review, we intend to elucidate the intricate dynamics surrounding cultural and linguistic barriers in mental health care.

**Results:**

Migrants from diverse backgrounds face many challenges, including the loss of homes, livelihoods, and family, often leading to mental health issues like depression, anxiety, and post-traumatic stress disorder. Two primary challenges include adapting to a new life and experiencing discrimination and marginalization (Amri et al., 2013; Satinsky et al., 2019). Social stigma and mistrust hinder access to mental health services. Limited culturally competent services widen the gap between mental health needs and help-seeking attitudes. Language barriers significantly contribute to disparities in access to services (Amri et al., 2013). Additionally, mental health care providers’ organizational culture often prioritizes ‘ideal’ patients who are native speakers, favouring individual-oriented treatment over community-focused care (Desai et al., 2021).

To address these barriers effectively, it is crucial to employ specific strategies. The Multi-Phase Model of Psychotherapy, Social Justice, and Human Rights (MPM) equips mental health counsellors to better serve immigrant communities while addressing social stigma. This comprehensive framework comprises five phases of intervention: psychoeducation, culturally responsive service delivery, cultural orientation, collaboration with local healers, and connecting patients to essential resources (Amri et al., 2013). Additionally, practitioners should receive training in effective collaboration with interpreters to provide multilingual healthcare (Brisset et al., 2014).

**Conclusions:**

Addressing cultural and linguistic barriers in mental health care is vital. The Multi-Phase Model of Psychotherapy offers a promising approach, while collaboration with interpreters remains essential.

**Disclosure of Interest:**

None Declared

